# Large language models to identify social determinants of health in electronic health records

**DOI:** 10.1038/s41746-023-00970-0

**Published:** 2024-01-11

**Authors:** Marco Guevara, Shan Chen, Spencer Thomas, Tafadzwa L. Chaunzwa, Idalid Franco, Benjamin H. Kann, Shalini Moningi, Jack M. Qian, Madeleine Goldstein, Susan Harper, Hugo J. W. L. Aerts, Paul J. Catalano, Guergana K. Savova, Raymond H. Mak, Danielle S. Bitterman

**Affiliations:** 1 Artificial Intelligence in Medicine (AIM) Program, Mass General Brigham, Harvard Medical School, Boston, MA USA; 2 Department of Radiation Oncology, Brigham and Women’s Hospital/Dana-Farber Cancer Institute, Boston, MA USA; 3 Computational Health Informatics Program, Boston Children’s Hospital, Harvard Medical School, Boston, MA USA; 4 Adult Resource Office, Dana-Farber Cancer Institute, Boston, MA USA; 5 Radiology and Nuclear Medicine, GROW & CARIM, Maastricht University, Maastricht, The Netherlands; 6 Department of Data Science, Dana-Farber Cancer Institute and Department of Biostatistics, Harvard T. H. Chan School of Public Health, Boston, MA USA

**Keywords:** Health care, Machine learning

## Abstract

Social determinants of health (SDoH) play a critical role in patient outcomes, yet their documentation is often missing or incomplete in the structured data of electronic health records (EHRs). Large language models (LLMs) could enable high-throughput extraction of SDoH from the EHR to support research and clinical care. However, class imbalance and data limitations present challenges for this sparsely documented yet critical information. Here, we investigated the optimal methods for using LLMs to extract six SDoH categories from narrative text in the EHR: employment, housing, transportation, parental status, relationship, and social support. The best-performing models were fine-tuned Flan-T5 XL for any SDoH mentions (macro-F1 0.71), and Flan-T5 XXL for adverse SDoH mentions (macro-F1 0.70). Adding LLM-generated synthetic data to training varied across models and architecture, but improved the performance of smaller Flan-T5 models (delta F1 + 0.12 to +0.23). Our best-fine-tuned models outperformed zero- and few-shot performance of ChatGPT-family models in the zero- and few-shot setting, except GPT4 with 10-shot prompting for adverse SDoH. Fine-tuned models were less likely than ChatGPT to change their prediction when race/ethnicity and gender descriptors were added to the text, suggesting less algorithmic bias (*p* < 0.05). Our models identified 93.8% of patients with adverse SDoH, while ICD-10 codes captured 2.0%. These results demonstrate the potential of LLMs in improving real-world evidence on SDoH and assisting in identifying patients who could benefit from resource support.

## Introduction

Health disparities have been extensively documented across medical specialties^1–3^. However, our ability to address these disparities remains limited due to an insufficient understanding of their contributing factors. Social determinants of health (SDoH), are defined by the World Health Organization as “the conditions in which people are born, grow, live, work, and age…shaped by the distribution of money, power, and resources at global, national, and local levels”^4^. SDoH may be adverse or protective, impacting health outcomes at multiple levels as they likely play a major role in disparities by determining access to and quality of medical care. For example, a patient cannot benefit from an effective treatment if they don’t have transportation to make it to the clinic. There is also emerging evidence that exposure to adverse SDoH may directly affect physical and mental health via inflammatory and neuro-endocrine changes^5–8^. In fact, SDoH are estimated to account for 80–90% of modifiable factors impacting health outcomes^9^.

SDoH are rarely documented comprehensively in structured data in the electronic health records (EHRs)^10–12^, creating an obstacle to research and clinical care. Instead, issues related to SDoH are most frequently described in the free text of clinic notes, which creates a bottleneck for incorporating these critical factors into databases to research the full impact and drivers of SDoH, and for proactively identifying patients who may benefit from additional social work and resource support.

Natural language processing (NLP) could address these challenges by automating the abstraction of these data from clinical texts. Prior studies have demonstrated the feasibility of NLP for extracting a range of SDoH^13–23^. Yet, there remains a need to optimize performance for the high-stakes medical domain and to evaluate state-of-the-art language models (LMs) for this task. In addition to anticipated performance changes scaling with model size, large LMs may support EHR mining via data augmentation. Across medical domains, data augmentation can boost performance and alleviate domain transfer issues and so is an especially promising approach for the nearly ubiquitous challenge of data scarcity in clinical NLP^24–26^. The advanced capabilities of state-of-the-art large LMs to generate coherent text open new avenues for data augmentation through synthetic text generation. However, the optimal methods for generating and utilizing such data remain uncertain. Large LM-generated synthetic data may also be a means to distill knowledge represented in larger LMs to more computationally accessible smaller LMs^27^. In addition, few studies assess the potential bias of SDoH information extraction methods across patient populations. LMs could contribute to the health inequity crisis if they perform differently in diverse populations and/or recapitulate societal prejudices^28^. Therefore, understanding bias is critical for future development and deployment decisions.

Here, we characterize optimal methods, including the role of synthetic clinical text, for SDoH extraction using large language models. Specifically, we develop models to extract six key SDoH: employment status, housing issues, transportation issues, parental status, and social support. We investigate the value of incorporating large LM-generated synthetic SDoH data during the fine-tuning stage. We assess the performance of large LMs, including GPT3.5 and GPT4, in zero- and few-shot settings for identifying SDoH, and we explore the potential for algorithmic bias in LM predictions. Our methods could yield real-world evidence on SDoH, assist in identifying patients who could benefit from resource and social work support, and draw attention to the under-documented impact of social factors on health outcomes.

## Results

### Model performance

Table 1 shows the performance of fine-tuned models for both SDoH tasks on the radiotherapy test set. The best-performing model for any SDoH mention task was Flan-T5 XXL (3 out of 6 categories) using synthetic data (Macro-F1 0.71). The best-performing model for the adverse SDoH mention task was Flan-T5 XL without synthetic data (Macro-F1 0.70). In general, the Flan-T5 models outperformed BERT, and model performance scaled with size. However, although the Flan-T5 XL and XXL models were the largest models evaluated in terms of total parameters because LoRA was used for their fine-tuning, the fewest parameters were tuned for these models: 9.5 M and 18 M for Flan-TX XL and XXL, respectively, compared to 110 M for BERT. The negative class generally had the best performance overall, followed by Relationship and Employment. Performance varied quite a bit across the models for the other classes.

**Table 1:** Model performance on the in-domain RT test dataset.

Any social determinant of health (SDoH)											
Model	Parameters (total/tuned)	Macro-F1			No SDoH (F1)	Employment (F1)	Housing (F1)	Parent (F1)	Relationship (F1)	Social support (F1)	Transportation (F1)
		Mean (95% CI)^a^	Delta F1^b^	*P* value							
BERT-base	110 M/110 M										
Gold data only		0.53 (0.46–0.59)	−0.06	<0.01	1.00	0.72	0.00	0.00	**0.96**	0.59	0.50
Gold + synthetic data		0.47 (0.44–0.52)			1.00	0.62	0.00	0.29	0.93	0.49	0.00
Flan-T5-base	250 M/250 M										
Gold data only		0.36 (0.34–0.39)	+0.13	<0.01	0.99	0.34	0.00	0.00	0.83	0.38	0.00
Gold + synthetic data		0.49 (0.40–0.60)			1.00	0.67	0.37	0.00	0.93	0.28	0.25
Flan-T5-large	780 M/780 M										
Gold data only		0.42 (0.40–0.45)	+0.18	<0.01	1.00	0.72	0.00	0.00	0.93	0.31	0.00
Gold + synthetic data		0.60 (0.50–0.68)			1.00	0.76	0.67	0.24	0.91	0.48	0.18
Flan-T5 XL	3B/9.5 M										
Gold data only		0.65 (0.54–0.73)	+0.03	<0.01	0.99	0.71	0.57	0.55	0.92	0.50	0.31
Gold + synthetic data		0.68 (0.59–0.76)			1.00	0.73	0.55	0.56	0.94	0.52	**0.53**
Flan-T5 XXL	11B/18 M										
Gold data only		0.65 (0.56–0.75)	+0.05	<0.01	1.00	0.76	0.33	**0.65**	0.95	0.51	0.44
Gold + synthetic data		**0.70 (0.60–0.77)**			1.00	**0.80**	**0.67**	0.47	0.93	**0.60**	0.47

Adverse Social Determinants of Health (SDoH)											
Model	Parameters (total/tuned)	Macro-F1			No SDoH (F1)	Employment (F1)	Housing (F1)	Parent (F1)	Relationship (F1)	Social support (F1)	Transportation (F1)
		Mean (95% CI)	Delta F1	*P* value							
BERT-base	110 M/110 M										
Gold data only		0.64 (0.55–0.73)	−0.09	<0.01	1.00	0.68	0.67	0.31	0.90	0.37	**0.60**
Gold + synthetic data		0.55 (0.45–0.67)			1.00	**0.75**	0.37	0.36	0.78	0.38	0.4
Flan-T5-base	250 M/250 M										
Gold data only		0.24 (0.18–0.31)	+0.11	<0.01	1.00	0.00	0.00	0.00	0.43	0.00	0.25
Gold + synthetic data		0.35 (0.26–0.45)			1.00	0.30	0.33	0.00	0.56	0.00	0.25
Flan-T5-large	780 M/780 M										
Gold data only		0.27 (0.23–0.31)	+0.22	<0.01	0.99	0.46	0.00	0.00	0.47	0.00	0.00
Gold + synthetic data		0.49 (0.40–0.59)			1.00	0.58	0.54	0.33	0.66	0.22	0.17
Flan-T5 XL	3B/9.5 M										
Gold data only		**0.69 (0.57–0.78)**	0.00	0.53	1.00	0.76	0.57	0.52	**0.93**	0.44	0.67
Gold + synthetic data		**0.69 (0.57–0.77)**			1.00	0.72	**0.67**	0.49	0.87	**0.56**	0.57
Flan-T5 XXL	11B/18 M										
Gold data only		0.63 (0.52–0.72)	+0.03	<0.01	1.00	0.67	0.50	**0.60**	0.91	0.31	0.45
Gold + synthetic data		0.66 (0.55–0.74)			1.00	0.62	0.60	0.55	0.89	0.53	0.46

The 95% CI for Macro-F1 is calculated by bootstrapping 3400 times (to achieve bootstrap SE < 0.01) with replacement. The SE of the 95% confidence interval limits is 0.0091, ascertained by performing bootstrapping 3,400 times on three distinct samples. Delta F1 score is the change in Macro-F1 when synthetic data are added to the fine-tuning data. Bolded text indicates the best performance with and without synthetic data augmentation. *p* values are computed with Mann–Whitney *U* test. *CI* confidence interval, *SE* standard error.

For both tasks, the best-performing models with synthetic data augmentation used sentences from both rounds of GPT3.5 prompting. Synthetic data augmentation tended to lead to the largest performance improvements for classes with few instances in the training dataset and for which the model trained on gold-only data had very low performance (Housing, Parent, and Transportation).

The performance of the best-performing models for each task on the immunotherapy and MIMIC-III datasets is shown in Table 2*.* Performance was similar in the immunotherapy dataset, which represents a separate but similar patient population treated at the same hospital system. We observed a performance decrement in the MIMIC-III dataset, representing a more dissimilar patient population from a different hospital system. Performance was similar between models developed with and without synthetic data.

**Table 2:** Results of the best-performing models on the out-of-domain test datasets.

Any social determinant of health (SDoH)										
Dataset	Macro-F1			No SDoH (F1)	Employment (F1)	Housing (F1)	Parent (F1)	Relationship (F1)	Social support (F1)	Transportation (F1)
	Mean (95% CI)	Delta F1	*P* value							
Immunotherapy										
FlanXXL: Gold data only	0.70 (0.63–0.76)	+0.01	<0.01	0.99	0.83	0.55	0.69	0.93	0.46	0.46
FlanXXL: Gold + synthetic data	0.71 (0.64–0.76)			0.99	0.79	0.55	0.68	0.91	0.63	0.40
MIMIC-III										
FlanXXL: Gold data only	0.57 (0.49–0.63)	−0.02	<0.01	0.98	0.65	0.00	0.63	0.91	0.32	0.50
FlanXXL: Gold + synthetic data	0.55 (0.49–0.61)			0.98	0.69	0.24	0.44	0.91	0.33	0.24

Adverse social determinants of health (SDoH)										
Dataset	Macro-F1			No SDoH (F1)	Employment (F1)	Housing (F1)	Parent (F1)	Relationship (F1)	Social support (F1)	Transportation (F1)
	Mean (95% CI)^a^	Delta F1^b^	*P* value							
Immunotherapy										
FlanXL: Gold data only	0.63 (0.54–0.72)	+0.03	<0.01	1.00	0.56	0.46	0.68	0.81	0.50	0.46
FlanXL: Gold + synthetic data	0.66 (0.58–0.72)			1.00	0.60	0.63	0.60	0.81	0.59	0.40
MIMIC-III										
FLANXL: Gold data only	0.53 (0.47–0.60)	−0.02	<0.01	0.99	0.51	0.50	0.53	0.65	0.22	0.20
FLANXL: Gold + synthetic data	0.51 (0.43–0.59)			0.99	0.55	0.35	0.54	0.68	0.43	0.20

The 95% CI for Macro-F1 is calculated by bootstrapping 3400 times (to achieve bootstrap SE < 0.01) with replacement. The SE of the 95% confidence interval limits is 0.0074, ascertained by performing bootstrapping 3400 times on three distinct samples. Delta F1 score is the change in Macro-F1 when synthetic data are added to the fine-tuning data. Bolded text indicates the best performance with and without synthetic data augmentation. *p* values are computed with Mann–Whitney *U* test. *CI* confidence interval, *SE* standard error.

### Ablation studies

**Fig. 1:**
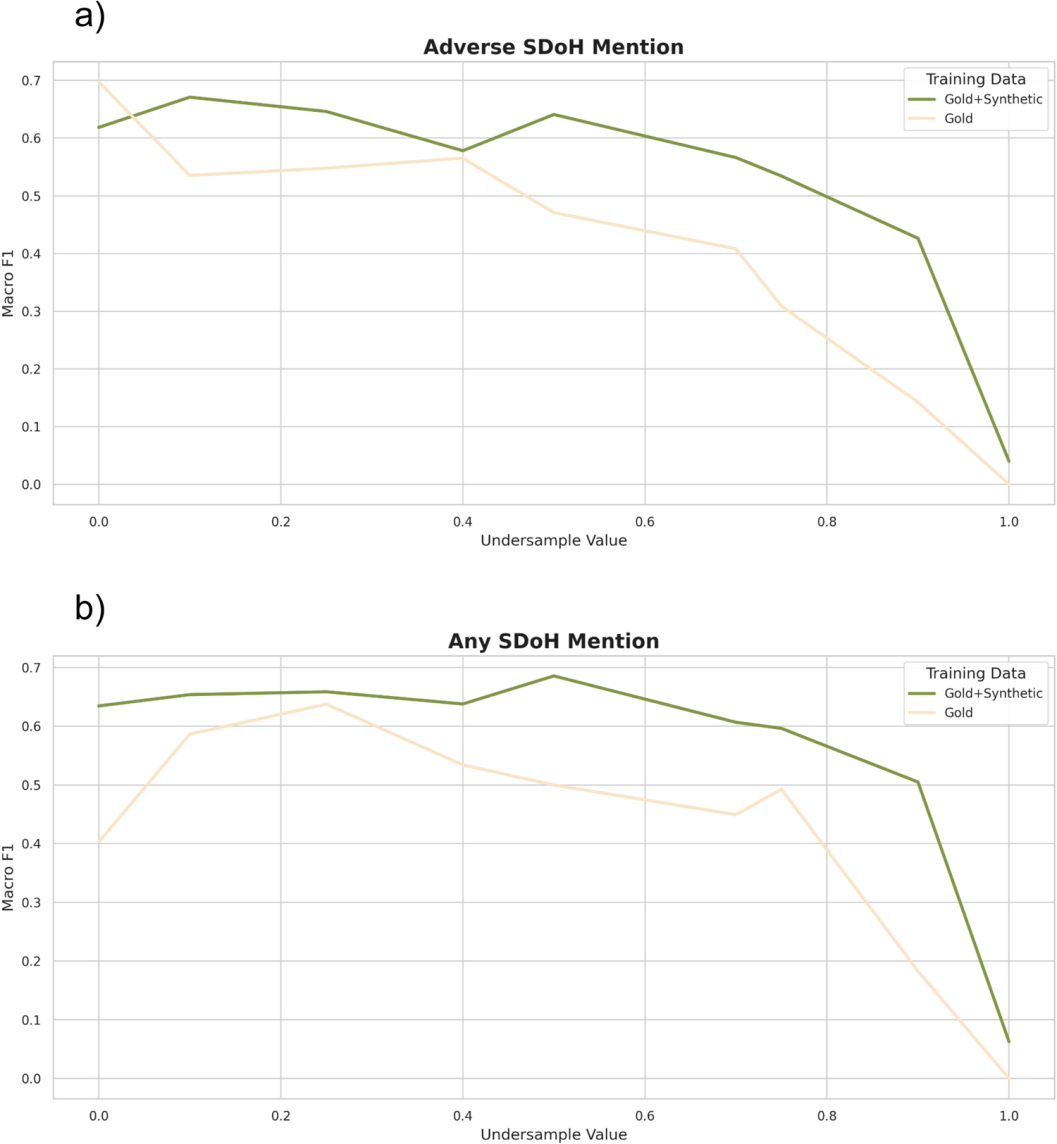
Ablation studies. Performance in Macro-F1 of Flan-T5 XL models fine-tuned using gold data only (orange line) and gold and synthetic data (green line), as gold-labeled sentences are gradually reduced by undersample value from the training dataset for the **a** adverse social determinant of health (SDoH) mention task and **b** any SDoH mention task. The full gold-labeled training set is comprised of 29,869 sentences, augmented with 1800 synthetic SDoH sentences, and tested on the in-domain RT test dataset. SDoH Social determinants of health.

The ablation studies showed a consistent deterioration in model performance across all SDoH tasks and categories as the volume of real gold SDoH sentences progressively decreased, although models that included synthetic data maintained performance at higher levels throughout and were less sensitive to decreases in gold data (Fig. 1, Supplementary Table 1). When synthetic data were included in the training, performance was maintained until ~50% of gold data were removed from the train set. Conversely, without synthetic data, performance dropped after about 10–20% of the gold data were removed from the train set, mimicking a true low-resource setting.

### Error analysis

The leading discrepancies between ground truth and model prediction for each task are in Supplementary Table 2. Qualitative analysis revealed 4 distinct error patterns: Human annotator error; false positives and false negatives for Relationship and Support labels in the presence of any family mentions that did not correlate with the correct label; incorrect labels due to information present in the note but external to the sentence and therefore not accessible to the model or that required implied/assumed knowledge; and incorrect labeling of a non-adverse SDoH as an adverse SDoH.

### ChatGPT-family model performance

**Fig. 2:**
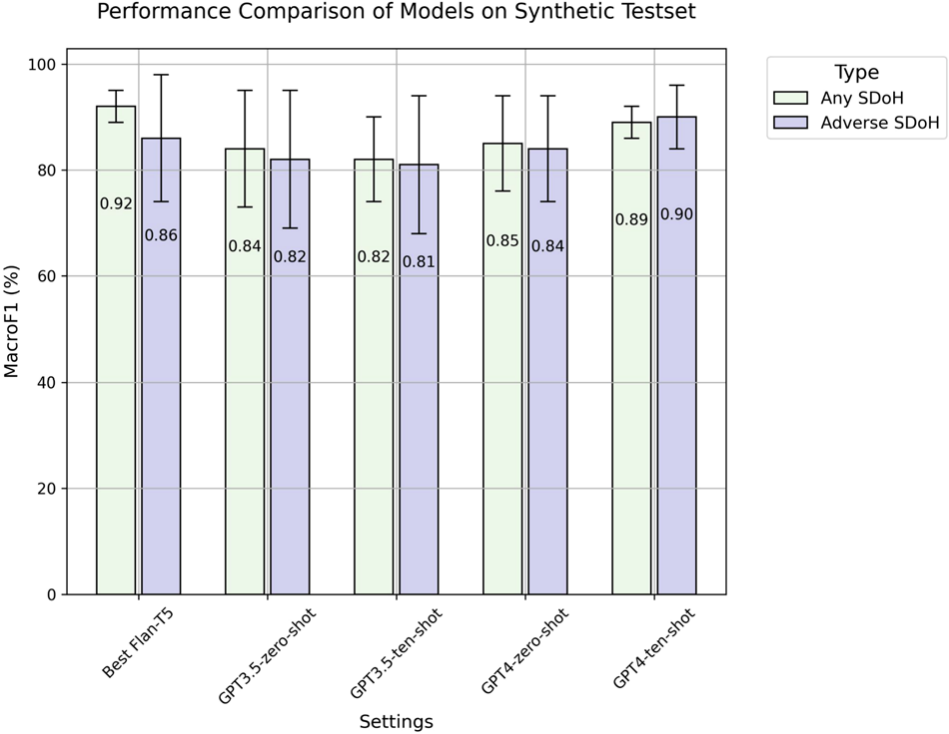
Fine-tuned LLMs versus ChatGPT-family models. Comparison of model performance between our fine-tuned Flan-T5 models against zero- and 10-shot GPT. Macro-F1 was measured using our manually validated synthetic dataset. The GPT-turbo-0613 version of GPT3.5 and the GPT4–0613 version of GPT4 were used. Error bars indicate the 95% confidence intervals. LLM large language model.

When evaluating our fine-tuned Flan-T5 models on the synthetic test dataset against GPT-turbo-0613 and GPT4–0613, our model surpassed the performance of the top-performing 10-shot learning GPT model by a margin of Macro-F1 0.03 on any SDoH task (*p* < 0.01), but fall shorts on adverse SDoH task (*p* < 0.01) (Table 3, Fig. 2).

**Table 3:** Model performance on synthetic test data.

Any social determinant of health (SDoH)								
Model parameters		Mean Macro-F1 (95% CI)	Employment (F1)	Housing (F1)	Parent (F1)	Relationship (F1)	Social support (F1)	Transportation (F1)
FT Flan-T5 XXL	11B	**0.92 (0.62–0.95)**	**0.92**	**0.91**	0.63	**0.95**	**0.77**	**0.93**
GPT3.5	175B							
Zero-shot		0.84 (0.48–0.95)	0.94	0.87	0.85	0.82	0.49	0.84
10-shot		0.82 (0.60–0.90)	0.89	0.89	0.76	0.79	0.61	0.85
GPT4	Unknown							
Zero-shot		0.85 (0.48–0.94)	0.94	0.83	0.72	0.88	0.49	0.86
10-shot		0.88 (0.58–0.93)	0.91	0.90	**0.96**	0.82	0.59	0.91

Adverse social determinants of health (SDoH)								
Model parameters		Mean Macro-F1 (95% CI)^a^	Employment (F1)	Housing (F1)	Parent (F1)	Relationship(F1)	Social support (F1)	Transportation (F1)
FT Flan-T5 XL	3B	0.86 (0.65–0.98)	0.86	0.86	0.65	**0.98**	**0.84**	0.86
GPT3.5	175B							
Zero-shot		0.82 (0.51–0.95)	0.77	0.93	0.87	0.72	0.52	0.94
10-shot		0.81 (0.50–0.94)	**0.93**	0.83	0.78	0.70	0.50	0.93
GPT4	Unknown							
Zero-shot		0.84 (0.52–0.94)	0.79	**0.94**	**0.94**	0.78	0.53	0.89
10-shot		**0.90 (0.71–0.96)**	0.92	0.91	0.90	0.73	0.73	**0.96**

The 95% CI (confidence interval) for Macro-F1 is calculated by bootstrapping 10000 times (to achieve bootstrap SE < 0.01) with replacement. The SE of the 95% confidence interval limits is 0.0038, ascertained by performing bootstrapping 10,000 times on three distinct samples. Bolded text indicates the best performance. *FT* fine-tuned, *CI* confidence interval, *SE* standard error.

### Language model bias evaluation

**Fig. 3:**
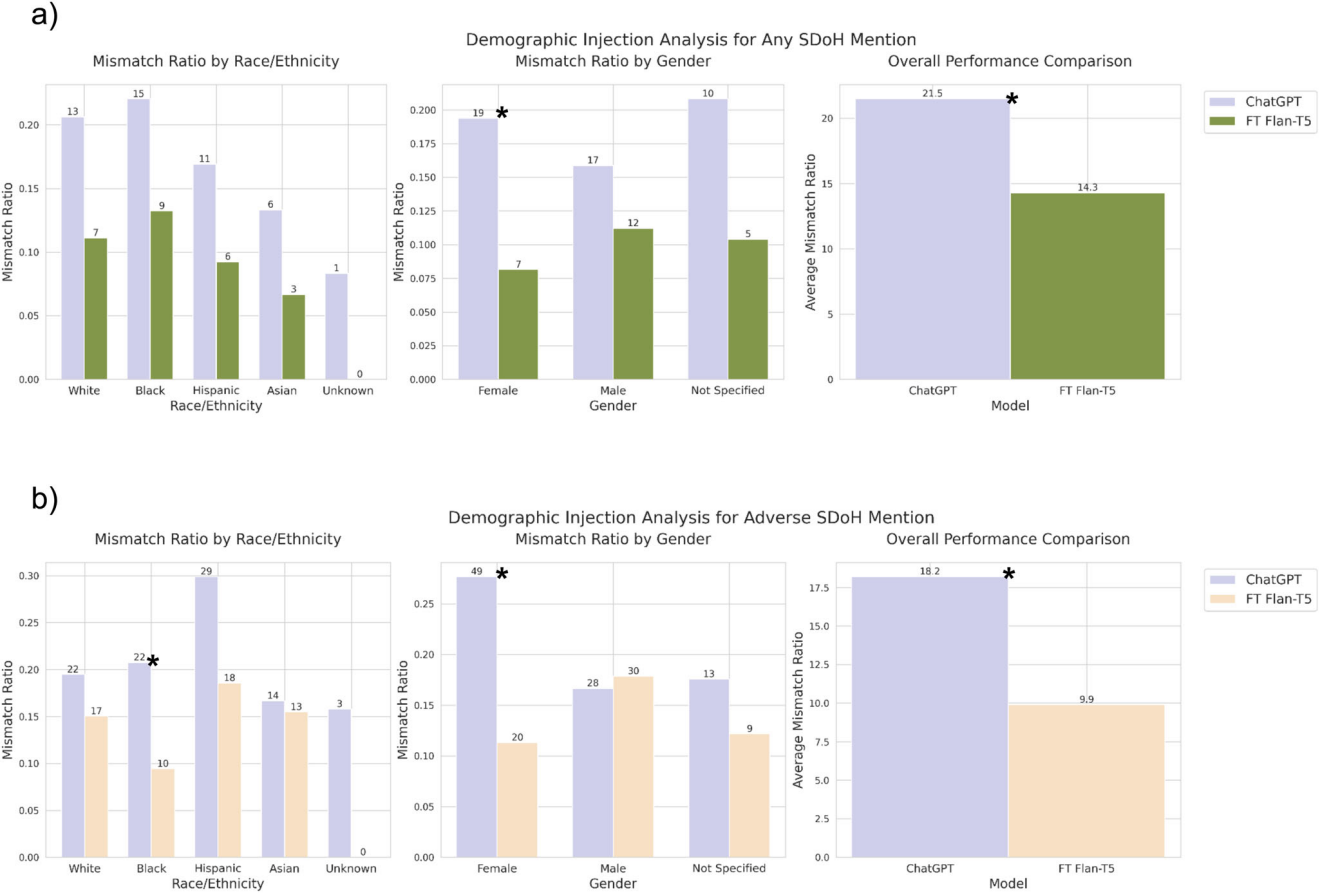
LLM bias evaluation. The proportion of synthetic sentence pairs with and without demographics injected led to a classification mismatch, meaning that the model predicted a different SDoH label for each sentence in the pair. Results are shown across race/ethnicity and gender for **a** any SDoH mention task and **b** adverse SDoH mention task. Asterisks indicate statistical significance (*P* ≤ 0.05) chi-squared tests for multi-class comparisons and 2-proportion *z* tests for binary comparisons. LLM large language model, SDoH Social determinants of health.

Both fine-tuned Flan-T5 models and ChatGPT provided discrepant classification for synthetic sentence pairs with and without demographic information injected (Fig. 3). However, the discrepancy rate of our fine-tuned models was nearly half that of ChatGPT: 14.3% vs. 21.5% of sentence pairs for any SDoH (*P* = 0.007) and 9.9% vs. 18.2% of sentence pairs for adverse SDoH (*P* = 0.005) for fine-tuned Flan-T5 vs. ChatGPT, respectively. ChatGPT was significantly more likely to change its classification when a female gender was injected compared to a male gender for the Any SDoH task (*P* = 0.01); no other within-model comparisons were statistically significant. Sentences gold-labeled as Support for both any SDoH and adverse SDoH mentions were most likely to lead to discrepant predictions for ChatGPT (56.3% (27/48)) and (21.0% (9/29)), respectively). Employment gold-labeled sentences were most likely to lead to discrepant prediction for any SDoH mention fine-tuned model (14.4% (13/90)), and Transportation for adverse SDoH mention fine-tuned model (12.2% (6/49)).

### Comparison with structured EHR data

Our best-performing models for any SDoH mention correctly identified 95.7% (89/93) patients with at least one SDoH mention, and 93.8% (45/48) patients with at least one adverse SDoH mention (Supplementary Tables 3 and 4). SDoH entered as structured Z-code in the EHR during the same timespan identified 2.0% (1/48) with at least one adverse SDoH mention (all mapped Z-codes were adverse) (Supplementary Table 5). Supplementary Figs. 1 and 2 show that patient-level performance when using model predictions out-performed Z-codes by a factor of at least 3 for every label for each task (Macro-F1 0.78 vs. 0.17 for any SDoH mention and 0.71 vs. 0.17 for adverse SDoH mention).

## Discussion

We developed multilabel classifiers to identify the presence of 6 different SDoH documented in clinical notes, demonstrating the potential of large LMs to improve the collection of real-world data on SDoH and support the appropriate allocation of resources support to patients who need it most. We identified a performance gap between a more traditional BERT classifier and larger Flan-T5 XL and XXL models. Our fine-tuned models outperformed ChatGPT-family models with zero- and few-shot learning for most SDoH classes and were less sensitive to the injection of demographic descriptors. Compared to diagnostic codes entered as structured data, text-extracted data identified 91.8% more patients with an adverse SDoH. We also contribute new annotation guidelines as well as synthetic SDoH datasets to the research community.

All of our models performed well at identifying sentences that do not contain SDoH mentions (F1 ≥ 0.99 for all). For any SDoH mentions, performance was worst for parental status and transportation issues. For adverse SDoH mentions, performance was worst for parental status and social support. These findings are unsurprising given the marked class imbalance for all SDoH labels—only 3% of sentences in our training set contained any SDoH mention. Given this imbalance, our models’ ability to identify sentences that contain SDoH language is impressive. In addition, these SDoH descriptions are semantically and linguistically complex. In particular, sentences describing social support are highly variable, given the variety of ways individuals can receive support from their social systems during care. Interestingly, our best-performing models demonstrated strong performance in classifying housing issues (Macro-F1 0.67), which was our scarcest label with only 20 instances in the training dataset. This speaks to the potential of large LMs in improved real-world data collection for very sparsely documented information, which is the most likely to be missed via manual review.

The recent advancements in large LMs have opened a pathway for synthetic text generation that may improve model performance via data augmentation and enable experiments that better protect patient privacy^29^. This is an emerging area of research that falls within a larger body of work on synthetic patient data across a range of data types and end-uses^30,31^. Our study is among the first to evaluate the role of contemporary generative large LMs for synthetic clinical text to help unlock the value of unstructured data within the EHR. We were particularly interested in synthetic clinical data as a means to address the aforementioned scarcity of SDoH documentation, and our findings may provide generalizable insights for the common clinical NLP challenge of class imbalance—many clinically important data are difficult to identify among the huge amounts of text in a patient’s EHR. We found variable benefits of synthetic data augmentation across model architecture and size; the strategy was most beneficial for the smaller Flan-T5 models and for the rarest classes where performance was dismal using gold data alone. Importantly, the ablation studies demonstrated that only approximately half of the gold-labeled dataset was needed to maintain performance when synthetic data was included in training, although synthetic data alone did not produce high-quality models. Of note, we aimed to understand whether synthetic data for augmentation could be automatically generated using ChatGPT-family models without additional human annotation, and so it is possible that manual gold-labeling could further enhance the value of these data. However, this would decrease the value of synthetic data in terms of reducing annotation effort.

Our novel approach to generating synthetic clinical sentences also enabled us to explore the potential for ChatGPT-family models, GPT3.5 and GPT4, for supporting the collection of SDoH information from the EHR. We found that fine-tuning LMs that are orders of magnitude smaller than ChatGPT-family models, even with our relatively small dataset, generally out-performed zero-shot and few-shot learning with ChatGPT-family models, consistent with prior work evaluating large LMs for clinical uses^32–34^. Nevertheless, these models showed promising performance given that they were not explicitly trained for clinical tasks, with the caveat that it is hard to make definite conclusions based on synthetic data. Additional prompt engineering could improve the performance of ChatGPT-family models, such as developing prompts that provide details of the annotation guidelines as done by Ramachandran et al.^34^. This is an area for future study, especially once these models can be readily used with real clinical data. With additional prompt engineering and model refinement, performance of these models could improve in the future and provide a promising avenue to extract SDoH while reducing the human effort needed to label training datasets.

It is well-documented that LMs learn the biases, prejudices, and racism present in the language they are trained on^35–38^. Thus, it is essential to evaluate how LMs could propagate existing biases, which in clinical settings could amplify the health disparities crisis^1–3^. We were especially concerned that SDoH-containing language may be particularly prone to eliciting these biases. Both our fine-tuned models and ChatGPT altered their SDoH classification predictions when demographics and gender descriptors were injected into sentences, although the fine-tuned models were significantly more robust than ChatGPT. Although not significantly different, it is worth noting that for both the fine-tuned models and ChatGPT, Hispanic and Black descriptors were most likely to change the classification for any SDoH and adverse SDoH mentions, respectively. This lack of significance may be due to the small numbers in this evaluation, and future work is critically needed to further evaluate bias in clinical LMs. We have made our paired demographic-injected sentences openly available for future efforts on LM bias evaluation.

SDoH are notoriously under-documented in existing EHR structured data^10–12,39^. Our findings that text-extracted SDoH information was better able to identify patients with adverse SDoH than relevant billing codes are in agreement with prior work showing under-utilization of Z-codes^10,11^. Most EMR systems have other ways to enter SDoH information as structured data, which may have more complete documentation, however, these did not exist for most of our target SDoH. Lyberger et al. evaluated other EHR sources of structured SDoH data and similarly found that NLP methods are a complementary source SDoH information extraction and were able to identify 10–30% of patients with tobacco, alcohol, and homelessness risk factors documented only in unstructured text^22^.

There have been several prior studies developing NLP methods to extract SDoH from the EHR^13–21,40^. The most common SDoH targeted in prior efforts include smoking history, substance use, alcohol use, and homelessness^23^. In addition, many prior efforts focus only on text in the Social History section of notes. In a recent shared task on alcohol, drug, tobacco, employment, and living situation event extraction from Social History sections, pre-trained LMs similarly provided the best performance^41^. Using this dataset, one study found that sequence-to-sequence approaches outperformed classification approaches, in line with our findings^42^. In addition to our technical innovations, our work adds to prior efforts by investigating SDoH which are less commonly targeted for extraction but nonetheless have been shown to impact healthcare^43–51^. We also developed methods that can mine information from full clinic notes, not only from Social History sections—a fundamentally more challenging task with a much larger class imbalance. Clinically-impactful SDoH information is often scattered throughout other note sections, and many note types, such as many inpatient progress notes and notes written by nurses and social workers, do not consistently contain Social History sections.

Our study has limitations. First, our training and out-of-domain datasets come from a predominantly white population treated at hospitals in Boston, Massachusetts, in the United States of America. This limits the generalizability of our findings. We could not exhaustively assess the many methods to generate synthetic data from ChatGPT. Instead, we chose to investigate prompting methods that could be easily reproduced by others and did not require extensive task-specific optimization, as this is likely not feasible for the many clinical NLP tasks for one may wish to generate synthetic data on. Incorporating real clinical examples in the prompt may improve the quality of the synthetic data and is an area of future research when large generative LMs become more widely available for use with protected health information and within the resource constraints of academic researchers and healthcare systems. Because we could not evaluate ChatGPT-family models using protected health information, our evaluations are limited to manually-verified synthetic sentences. Thus, our reported performance may not completely reflect true performance on real clinical text. Because the synthetic sentences were generated using ChatGPT itself, and ChatGPT presumably has not been trained on clinical text, we hypothesize that, if anything, performance would be worse on real clinical data. Finally, our models can only be as good as the annotated corpus. SDoH annotation is challenging due to its conceptually complex nature, especially for the Support tag, and labeling may also be subject to annotator bias^52^, all of which may impact ultimate performance.

Our findings highlight the potential of large LMs to improve real-world data collection and identification of SDoH from the EHR. In addition, synthetic clinical text generated by large LMs may enable better identification of rare events documented in the EHR, although more work is needed to optimize generation methods. Our fine-tuned models were less prone to bias than ChatGPT-family models and outperformed for most SDoH classes, especially any SDoH mentions, despite being orders of magnitude smaller. In the future, these models could improve our understanding of drivers of health disparities by improving real-world evidence and could directly support patient care by flagging patients who may benefit most from proactive resource and social work referral.

## Methods

### Data

Table 4 describes the patient populations of the datasets used in this study. Gender and race/ethnicity data and descriptors were collected from the EHR. These are generally collected either directly from the patient at registration, or by a provider, but the mode of collection for each data point was not available. Our primary dataset consisted of a corpus of 800 clinic notes from 770 patients with cancer who received radiotherapy (RT) at the Department of Radiation Oncology at Brigham and Women’s Hospital/Dana-Farber Cancer Institute in Boston, Massachusetts, from 2015 to 2022. We also created two out-of-domain test datasets. First, we collected 200 clinic notes from 170 patients with cancer treated with immunotherapy at Dana-Farber Cancer, and not present in the RT dataset. Second, we collected 200 notes from 183 patients in the MIMIC (Medical Information Mart for Intensive Care)-III database^53–55^, which includes data associated with patients admitted to the critical care units at Beth Israel Deaconess Medical Center in Boston, Massachusetts from 2001 to 2008. This study was approved by the Mass General Brigham institutional review board, and consent was waived as this was deemed exempt from human subjects research.

**Table 4:** Patient demographics across datasets.

Patients	Radiotherapy (in-domain) dataset				Out-of-domain validation datasets			
	Total (*n* = 770)	Train Set (*n* = 462)	Development set (*n* = 154)	Test set (*n* = 154)	Immunotherapy (*n* = 170)	MIMIC-III (*n* = 183)	Synthetic Validated (*n* = 480)	Synthetic Demo (*n* = 419)
Gender								
Male	344 (44.7%)	210 (45.5%)	70 (45.5%)	64 (41.6%)	75 (44.1%)	101 (55.2%)	N/A	168 (40.1%)
Female	426 (55.3%)	252 (54.5%)	84 (54.5%)	90 (58.4%)	95 (55.9%)	82 (44.8%)	N/A	177 (42.2%)
Not specified	0	0	0	0	0	0	N/A	74 (17.7%)
Race								
White	664 (86.2%)	396 (85.7%)	134 (87.0%)	134 (87.0%)	137 (80.6%)	132 (72.1%)	N/A	113 (26.9%)
Asian	21 (2.7%)	11 (2.4%)	6 (3.9%)	4 (2.6%)	9 (5.3%)	5 (2.7%)	N/A	106 (21.6%)
Black	37 (4.8%)	24 (5.2%)	5 (3.2%)	8 (5.2%)	11 (6.5%)	16 (8.7%)	N/A	84 (25.7%)
Two or more	3 (0.4%)	2 (0.4%)	0	1 (0.6%)	0	3 (1.6%)	N/A	0
Others	25 (3.2%)	17 (3.7%)	5 (3.2%)	3 (1.9%)	10 (5.9%)	1 (0.6%)	N/A	97 (23.2%)
Unknown	20 (2.6%)	12 (2.6%)	4 (2.6%)	4 (2.6%)	3 (1.8%)	25 (13.7%)	N/A	19 (4.5%)
Ethnicity								
Non-Hispanic	682 (88.6%)	420 (90.9%)	130 (84.4%)	132 (85.7%)	160 (94.1%)	158 (86.3%)	N/A	322 (76.8%)
Hispanic	11 (1.4%)	8 (1.7%)	2 (1.3%)	1 (0.6%)	20 (5.9%)	11 (6.0%)	N/A	97 (23.2%)
Unknown	77 (10.0%)	34 (7.4%)	22 (14.3%)	21 (13.6%)	0	14 (7.7%)	N/A	0

All data presented as *n* (%) unless otherwise noted. Synthetic Validated are the sentences used to evaluate GPT models, thus, there is no demographic information for this dataset. Synthetic Demo is the sentence used for bias evaluation, where demographic descriptors were inserted. *N/A* not applicable.

Only notes written by physicians, physician assistants, nurse practitioners, registered nurses, and social workers were included. To maintain a minimum threshold of information, we excluded notes with fewer than 150 tokens across all provider types. This helped ensure that the selected notes contained sufficient textual content. For notes written by all providers save social workers, we excluded notes containing any section longer than 500 tokens to avoid excessively lengthy sections that might have included less relevant or redundant information. For physician, physician assistant, and nurse practitioner notes, we used a customized medSpacy^56,57^ sectionizer to include only notes that contained at least one of the following sections: Assessment and Plan, Social History, and History/Subjective.

In addition, for the RT dataset, we established a date range, considering notes within a window of 30 days before the first treatment and 90 days after the last treatment. Additionally, in the fifth round of annotation, we specifically excluded notes from patients with zero social work notes. This decision ensured that we focused on individuals who had received social work intervention or had pertinent social context documented in their notes. For the immunotherapy dataset, we ensured that there was no patient overlap between RT and immunotherapy notes. We also specifically selected notes from patients with at least one social work note. To further refine the selection, we considered notes with a note date one month before or after the patient’s first social work note after it. For the MIMIC-III dataset, only notes written by physicians, social workers, and nurses were included for analysis. We focused on patients who had at least one social work note, without any specific date range criteria.

Prior to annotation, all notes were segmented into sentences using the syntok^58^ sentence segmenter as well as split into bullet points “•”. This method was used for all notes in the radiotherapy, immunotherapy, and MIMIC datasets for sentence-level annotation and subsequent classification.

### Task definition and data labeling

We defined our label schema and classification tasks by first carrying out interviews with subject matter experts, including social workers, resource specialists, and oncologists, to determine SDoH that are clinically relevant but not readily available as structured data in the EHR, especially as dynamic features over time. After initial interviews, a set of exploratory pilot annotations was conducted on a subset of clinical notes and preliminary annotation guidelines were developed. The guidelines were then iteratively refined and finalized based on the pilot annotations and additional input from subject matter experts. The following SDoH categories and their attributes were selected for inclusion in the project: Employment status (employed, unemployed, underemployed, retired, disability, student), Housing issue (financial status, undomiciled, other), Transportation issue (distance, resource, other), Parental status (if the patient has a child under 18 years old), Relationship (married, partnered, widowed, divorced, single), and Social support (presence or absence of social support).

We defined two multilabel sentence-level classification tasks:

1. Any SDoH mentions: The presence of language describing an SDoH category as defined above, regardless of the attribute.
2. Adverse SDoH mentions: The presence or absence of language describing an SDoH category with an attribute that could create an additional social work or resource support need for patients:

- **Employment status**: unemployed, underemployed, disability
- **Housing issue**: financial status, undomiciled, other
- **Transportation issue**: distance, resources, other
- **Parental status**: having a child under 18 years old
- **Relationship**: widowed, divorced, single
- **Social support**: absence of social support

After finalizing the annotation guidelines, two annotators manually annotated the RT corpus. In total, ten thousand one hundred clinical notes were annotated line-by-line using the annotation software Multi-document Annotation Environment (MAE v2.2.13)^59^. A total of 300/800 (37.5%) of the notes underwent dual annotation by two data scientists across four rounds. After each round, the data scientists and an oncologist performed discussion-based adjudication. Before adjudication, dually annotated notes had a Krippendorf’s alpha agreement of 0.86 and Cohen’s Kappa of 0.86 for any SDoH mention categories. For adverse SDoH mentions, notes had a Krippendorf’s alpha agreement of 0.76 and Cohen’s Kappa of 0.76. Detailed agreement metrics are in Supplementary Tables 6 and 7. A single annotator then annotated the remaining radiotherapy notes, the immunotherapy dataset, and the MIMIC-III dataset. Table 5 describes the distribution of labels across the datasets.

**Table 5:** Distribution of documents and sentence labels in each dataset.

Number of documents							
	Radiotherapy			Immunotherapy	MIMIC-III	Synthetic validated	Synthetic demo
	Train set	Development set	Test set				
Documents	481	160	159	200	200	N/A	N/A

Number of sentences–any SDoH mentions							
	Radiotherapy			Immunotherapy (*n* = 14,761)	MIMIC-III (*n* = 5328)	Synthetic validated (*n* = 480)	Synthetic demo (*n* = 419)
Label	Train set (*n* = 29,869)	Development set (*n* = 10,712)	Test set (*n* = 10,860)				
No SDoH	28992 (97.1%)	10429 (97.4%)	10582 (97.4%)	14319 (97.0%)	4968 (93.2%)	N/A	N/A
Employment	218 (0.7%)	65 (0.6%)	64 (0.6%)	103 (0.7%)	70 (1.3%)	136 (28.3%)	132 (31.5%)
Housing	20 (0.1%)	7 (0.1%)	4 (0.0%)	13 (0.1%)	3 (0.1%)	69 (14.4%)	64 (15.3%)
Parent	53 (0.2%)	24 (0.2%)	22 (0.2%)	30 (0.2%)	27 (0.5%)	67 (14.0%)	43 (10.3%)
Relationship	464 (1.6%)	153 (1.4%)	158 (1.5%)	241 (1.6%)	180 (3.4%)	152 (31.7%)	134 (32.0%)
Social Support	234 (0.8%)	51 (0.5%)	61 (0.6%)	86 (0.6%)	122 (2.3%)	102 (21.3%)	90 (21.5%)
Transportation	41 (0.1%)	13 (0.1%)	6 (0.1%)	25 (0.2%)	3 (0.1%)	61 (12.7%)	58 (13.8%)

Number of sentences–adverse SDoH mentions							
	Radiotherapy			Immunotherapy (*n* = 14,761)	MIMIC-III (*n* = 5328)	Synthetic validated (*n* = 289)	Synthetic demo (*n* = 253)
Label	Train Set (*n* = 29,869)	Development set (*n* = 10,712)	Test set (*n* = 10,860)				
No Adverse SDoH	29550 (98.9%)	10615 (99.1%)	10761 (99.1%)	14621 (99.1%)	5213 (97.8%)	N/A	N/A
Employment	93 (0.3%)	23 (0.2%)	30 (0.3%)	37 (0.3%)	39 (0.7%)	40 (13.8%)	39 (15.4%)
Housing	20 (0.1%)	7 (0.1%)	4 (0.0%)	13 (0.1%)	3 (0.1%)	69 (23.9%)	64 (25.3%)
Parent	53 (0.2%)	24 (0.2%)	22 (0.2%)	30 (0.2%)	27 (0.5%)	67 (23.2%)	43 (17.0%)
Relationship	86 (0.3%)	27 (0.3%)	31 (0.3%)	30 (0.2%)	23 (0.4%)	68 (23.5%)	62 (24.5%)
Social support	54 (0.2%)	8 (0.1%)	12 (0.1%)	12 (0.1%)	27 (0.5%)	39 (13.5%)	43 (17.0%)
Transportation	41 (0.1%)	13 (0.1%)	6 (0.1%)	25 (0.2%)	3 (0.1%)	61 (21.1%)	58 (22.9%)

All data presented as *n* (%) unless otherwise noted. Synthetic Validated are the sentences used to evaluate GPT models, thus, there is no demographic information for this dataset. Synthetic Demo is the sentence used for bias evaluation, where demographic descriptors were inserted. Labels sum to >100% because some sentences had more than 1 SDoH label. *SDoH* social determinants of health, *N/A* not applicable.

The annotation/adjudication team was composed of one board-certified radiation oncologist who completed a postdoctoral fellowship in clinical natural language processing, a Master’s-level computational linguist with a Bachelor’s degree in linguistics and 1-year prior experience working specifically with clinical text, and a Master’s student in computational linguistics with a Bachelor’s degree in linguistics. The radiation oncologist and Master’s level computational linguist led the development of the annotation guidelines, and trained the Master’s student in SDoH annotation over a period of 1 month via review of the annotation guidelines and iterative review of pilot annotations. During adjudication, if there was still ambiguity, we discussed with the two Resource Specialists on the research team to provide input in adjudication.

### Data augmentation

We employed synthetic data generation methods to assess the impact of data augmentation for the positive class, and also to enable an exploratory evaluation of proprietary large LMs that could not be downloaded locally and thus cannot be used with protected health information. In round 1, GPT-turbo-0301(ChatGPT) version of GPT3.5 via the OpenAI^60^ API was prompted to generate new sentences for each SDoH category, using sentences from the annotation guidelines as references. In round 2, in order to generate more linguistic diversity, the sample synthetic sentences output from round 1 were taken as references to generate another set of synthetic sentences. One-hundred sentences per category were generated in each round. Supplementary Table 8 shows the prompts for each sentence label type.

### Synthetic test set generation

Iteration 1 for generating SDoH sentences involved prompting the 538 synthetic sentences to be manually validated to evaluate ChatGPT, which cannot be used with protected health information. Of these, after human review only 480 were found to have any SDoH mention, and 289 to have an adverse SDoH mention (Table 5). For all synthetic data generation methods, no real patient data were used in prompt development or fine-tuning.

### Model development

The radiotherapy corpus was split into a 60%/20%/20% distribution for training, development, and testing respectively. The entire immunotherapy and MIMIC-III corpora were held-out for out-of-domain tests and were not used during model development.

The experimental phase of this study focused on investigating the effectiveness of different machine learning models and data settings for the classification of SDoH. We explored one multilabel BERT model as a baseline, namely bert-base-uncased^61^, as well as a range of Flan-T5 models^62,63^ including Flan-T5 base, large, XL, and XXL; where XL and XXL used a parameter efficient tuning method (low-rank adaptation (LoRA)^64^). Binary cross-entropy loss with logits was used for BERT, and cross-entropy loss for the Flan-T5 models. Given the large class imbalance, non-SDoH sentences were undersampled during training. We assessed the impact of adding synthetic data on model performance. Details on model hyper-parameters are in Supplementary Methods.

For sequence-to-sequence models, input consisted of the input sentence with “summarize” appended in front, and the target label (when used during training) was the text span of the label from the target vocabulary. Because the output did not always exactly correspond to the target vocabulary, we post-processed the model output, which was a simple split function on “,” and dictionary mapping from observed miss-generation e.g., “RELAT → RELATIONSHIP”. Examples of this label resolution are in Supplementary Methods.

### Ablation studies

Ablation studies were carried out to understand the impact of manually labeled training data quantity on performance when synthetic SDoH data is included in the training dataset. First, models were trained using 10%, 25%, 40%, 50%, 70%, 75%, and 90% of manually labeled sentences; both SDoH and non-SDoH sentences were reduced at the same rate. The evaluation was on the RT test set.

### Evaluation

During training and fine-tuning, we evaluated all models using the RT development set and assessed their final performance using bootstrap sampling of the held-out RT test set. Bootstrap sample number and size were calculated to achieve a precision level for the standard error of macro F1 of ±0.01. The mean and 95% confidence intervals from the bootstrap samples were calculated from the resulting bootstrap samples. We also sampled to ensure that our standard error on the 95% confidence interval limits was <0.01 as follows: Our selected bootstrap sample size matched the test data size, sampling with replacement. We then computed the 5th and 95th percentile values for each of the calculated k samples from the resulting distributions. The standard deviation of these percentile values was subsequently determined to establish the precision of the confidence interval limits. Examples of the bootstrap sampling calculations are in Supplementary Methods.

For each classification task, we calculated precision/positive predictive value, recall/sensitivity, and F1 (harmonic mean of recall and precision) as follows:

- Precision = TP/(TP + FP)
- Recall = TP/(TP + FN)
- F1 = (2*Precision*Recall)/(Precision+Recall)
- TP = true positives, FP = false positives, FN = false negatives

Manual error analysis was conducted on the radiotherapy dataset using the best-performing model.

### ChatGPT-family model evaluation

**Fig. 4:**
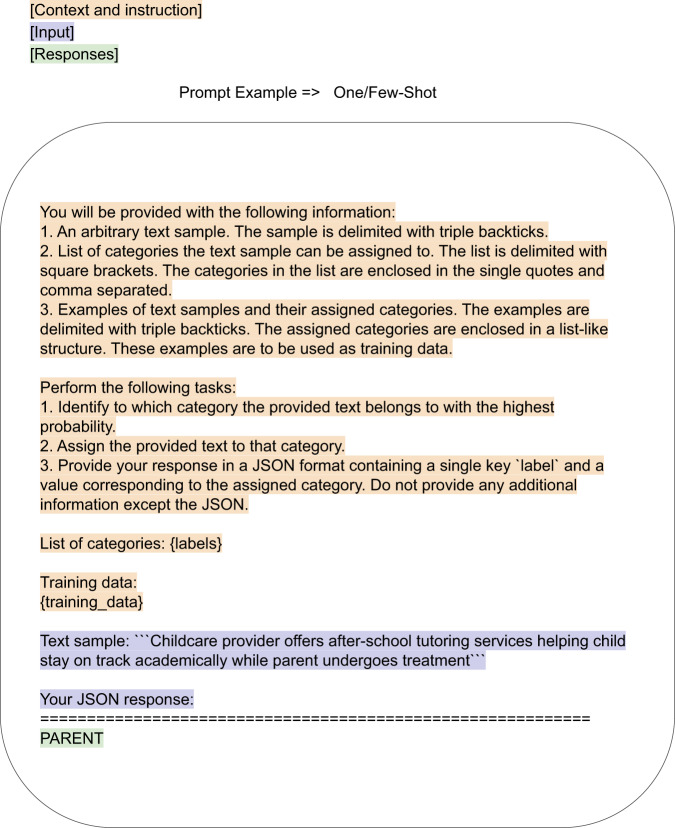
Prompting methods. Example of prompt templates used in the SKLLM package for GPT-turbo-0301 (GPT3.5) and GPT4 with temperature 0 to classify our labeled synthetic data. {labels} and {training_data} were sampled from a separate synthetic dataset, which was not human-annotated. The final label output is highlighted in green.

To evaluate ChatGPT, the Scikit-LLM^65^ multilabel zero-shot classifier and few-shot binary classifier were adapted to form a multilabel zero- and few-shot classifier (Fig. 4). A subset of 480 synthetic sentences whose labels were manually validated, were used for testing. Test sentences were inserted into the following prompt template, which instructs ChatGPT to act as a multilabel classifier model, and to label the sentences accordingly:

“Sample input: [TEXT]

Sample target: [LABELS]**”**

[TEXT] was the exemplar from the development/exemplar set.

[LABELS] was a comma-separated list of the labels for that exemplar, e.g. PARENT,RELATIONSHIP.

Of note, because we were unable to generate high-quality synthetic non-SDoH sentences, these classifiers did not include a negative class. We evaluated the most current ChatGPT model freely available at the time of this work, GPT-turbo-0613, as well as GPT4–0613, via the OpenAI API with temperature 0 for reproducibility.

### Language model bias evaluation

**Fig. 5:**
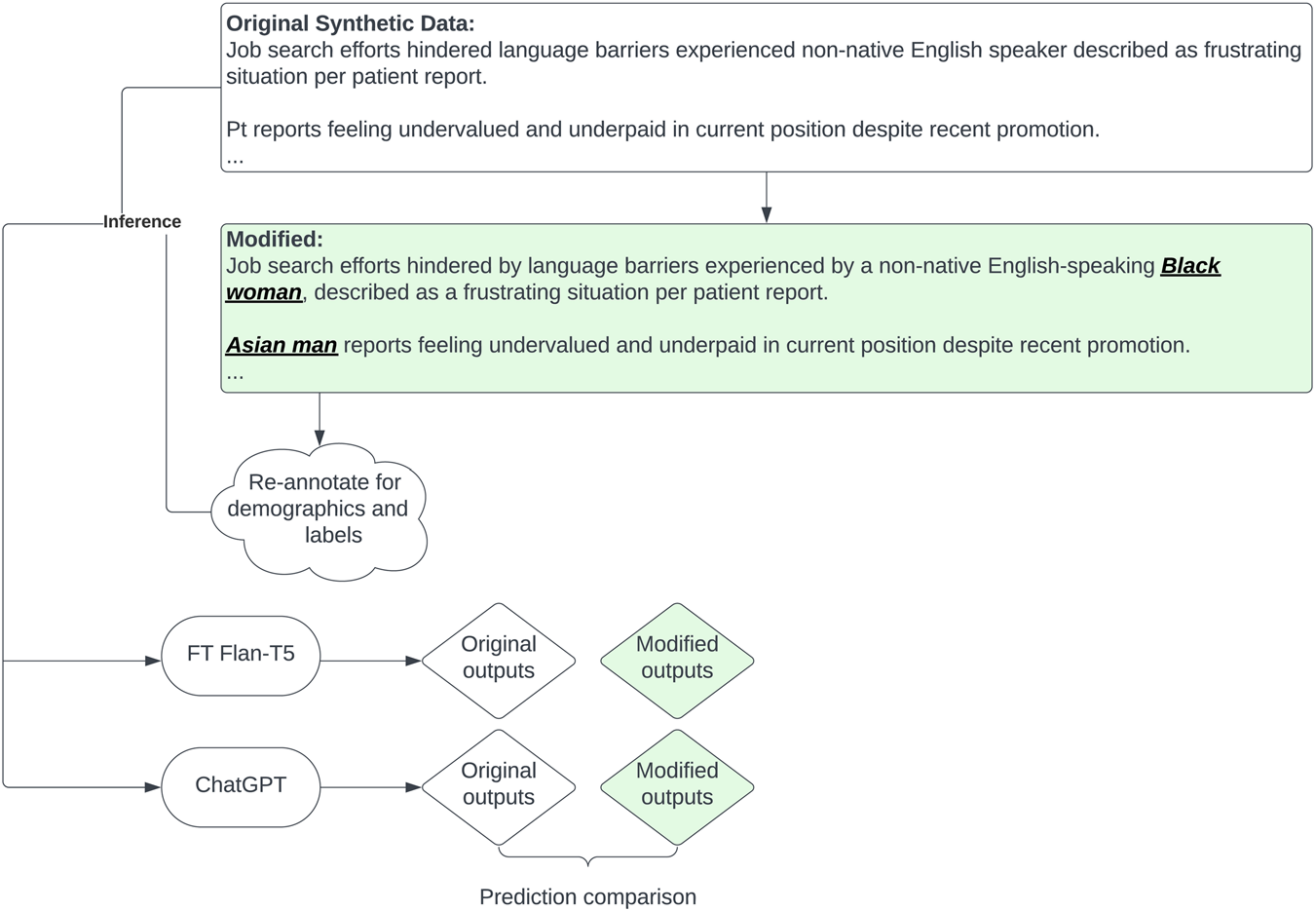
Demographic-injected SDoH language development. Illustration of generating and comparing synthetic demographic-injected SDoH language pairs to assess how adding race/ethnicity and gender information into a sentence may impact model performance. FT fine-tuned, SDoH Social determinants of health.

In order to test for bias in our best-performing models and in large LMs pre-trained on general text, we used GPT4 to insert demographic descriptors into our synthetic data, as illustrated in Fig. 5. GPT4 was supplied with our synthetically generated test sentences, and prompted to insert demographic information into them. For example, a sentence starting with “Widower admits fears surrounding potential judgment…” might become “Hispanic widower admits fears surrounding potential judgment…”. The prompt was as follows (in a batch of 10 ensure demographic variations):

“role”: “user”, “content”: **“**[ORIGINAL SENTENCE]\n swap the sentences patients above to one of the race/ethnicity [Asian, Black, white, Hispanic] and gender, and put the modified race and gender in bracket at the beginning like this \n Owner operator food truck selling gourmet grilled cheese sandwiches around town => \n [Asian female] Asian woman owner operator of a food truck selling gourmet grilled cheese sandwiches around town”

[ORIGINAL SENTENCE] was a sentence from a selected subset of our GPT3.5-generated synthetic data

These sentences were then manually validated; 419 had any SDoH mention, and 253 had an adverse SDoH mention.

### Comparison with structured EHR data

To assess the completeness of SDoH documentation in structured versus unstructured EHR data, we collected Z-codes for all patients in our test set. Z-codes are SDoH-related ICD-10-CM diagnostic codes, mapped most closely with our SDoH categories present as structured data for the radiotherapy dataset (Supplementary Table 9). Text-extracted patient-level SDoH information was defined as the presence of one or more labels in any note. We compared these patient-level labels to structured Z-codes entered in the EHR during the same time frame.

### Statistical analysis

Macro-F1 performance for each model type was compared when developed with or without synthetic data and for the ChatGPT-family model comparisons using the Mann–Whitney *U* test. The rate of discrepant SDoH classifications with and without the injection of demographic information was compared between the best-performing fine-tuned models and ChatGPT using chi-squared tests for multi-class comparisons and 2-proportion *z* tests for binary comparisons. A two-sided *P* ≤ 0.05 was considered statistically significant. Statistical analyses were carried out using the statistical Python package in scipy (Scipy.org). Python version 3.9.16 (Python Software Foundation) was used to carry out this work.

### Supplementary information

Supplemental Material

## Data Availability

The RT and immunotherapy datasets cannot be shared for the privacy of the individuals whose data were used in this study. All synthetic datasets used in this study are available at: https://github.com/AIM-Harvard/SDoH. The annotated MIMIC-III dataset is available after completion of a data use agreement at: 10.13026/6149-mb25^66^. The demographic-injected paired sentence dataset is available at: https://huggingface.co/datasets/m720/SHADR^67^.

## Code availability

The final annotation guidelines and all synthetic datasets used in this study are available at: https://github.com/AIM-Harvard/SDoH.
