# Supplementary material for: Large language models to identify social determinants of health in electronic health records
Source: NPJ Digit Med. 2024 Jan 11;7:6. doi: 10.1038/s41746-023-00970-0 (PMC10781957; doi:10.1038/s41746-023-00970-0)
Supplement: Supplementary file 1 — Supplemental Material [file 41746_2023_970_MOESM1_ESM.docx]

**Supplementary Methods**

*Annotation Details*

The most common type of disagreement between annotators involved one annotator labeling a sentence with a common tag (Support, Employment, and Relationship) and the other annotator not giving that sentence any label. For Employment and Relationship tags, this was most often due to simple annotation errors that get resolved through the adjudication process without much discussion. There were some instances for Employment where the language was vague as to whether the patient was on a break from work due to their job or whether they were no longer employed, which was a source of disagreements. The Support tag was a more conceptually complex tag resulting in disagreement. For example, there were disagreements based on whether a patient reporting feeling better because they had individuals visit their house, without details of the visit, consisted of support. Although it was still among the most prevalent of annotation disagreements, we believe the support tag’s complexity was minimized through pilot annotation rounds and guideline revisions. The disagreement types with >5 instances are listed below:

*Disagreement type Count*

NO_SDOH, SUPPORT_plus 27

NO_SDOH, EMPLOYMENT_employed 16

NO_SDOH, RELATIONSHIP_married 15

NO_SDOH, PARENT 8

NO_SDOH SUPPORT_minus 6

*Hyper-parameters for model training*

During the model development, the fine-tuning process of the best-performing models follows specific hyper-parameters using two *Nvidia RTX 3090 GPUs*. A per-device training batch size of 32 is used along with a learning rate of 1e-3. The model is trained for a total of 3 epochs. Additionally, the LoRA configuration is employed with a rank 'r' of 16 and a 'lora_alpha' value of 32. This configuration targets the "q" and "v" modules in the transformer layers and incorporates a dropout rate of 0.05.

per_device_train_batch_size=32,

learning_rate=1e-3,

num_train_epochs=3,

lora_config = LoraConfig(

r=16,

lora_alpha=32,

target_modules=["q", "v"],

lora_dropout=0.05,

bias="none",

task_type=TaskType.SEQ_2_SEQ_LM

)

*Label resolution examples for sequence-to-sequence models*

*Example 1:*

Source: “summarize: This is a patient with a history of heart disease.”

Target label: ‘<NO_SDOH>’

Output from model: “NO_SD”

Post-processed Output: [“<NO_SDOH>”]

*Example 2:*

Source: “summarize: The patient’s wife drives him to treatment every day.”

Target label: ‘RELATIONSHIP,SUPPORT’

Output from model: “RELAT,SUPPORT”

Post-processed Output: [“RELATIONSHIP”, “SUPPORT”]

*Bootstrap Sampling Calculations Example*

1. Set our desired precision level for standard error (SE) for the performance metric of macro-F1 to be +/- 0.01

2. Estimate variability by taking a small number of initial samples, e.g.:

- Take 100 initial bootstrap samples of size k = 10,860 (full test set size) with replacement.
- Calculate the standard deviation (σ) of F1 scores across these 100 bootstraps across all model pairs between gold-only data and gold+augmented data.
- Let's say σ is estimated to be 0.03.

3. Use the following formula to solve for n: SE = σ/√n

- For example, say σ was estimated to be 0.03:
  - 0.01 = 0.03/√n
  - n = 9 *100
- In practice, n varied from 2 to 34 across models, therefore we picked the largest (n=3400) for all comparison pairs.

4. Therefore, the recommended numbers to achieve our desired SE for macro-F1:

- n = 3400 (number of bootstrap samples)
- k = 10,860 (size of each bootstrap sample)

We calculated the mean and 95% confidence intervals from the 3400 bootstrap samples, and compared the difference in macro-F1 when adding augmented data using the Mann-Whitney U-test.

We established our metric as Macro F1, and sampled to ensure that our SE on the 95% confidence interval limits was < 0.01. Our selected bootstrap sample size matched the test-data size, sampling with replacement. We then computed the 5th and 95th percentile values for each of the calculated k samples from the resulting distributions. The standard deviation of these percentile values was subsequently determined to establish the precision of the confidence interval limits. For example, utilizing this methodological approach, a 95% confidence interval, accounting for a maximum variation of 5% and 95%, was ascertained to be 0.0091 for Table 3. This was validated through the execution of bootstrapping, performed 3,400 times across three distinct samples.

**Supplementary Tables**

**Supplementary Table 1.** Ablation studies of removing gold-labeled sentences in training

| **Any SDoH** | | | | | | | | |
| --- | --- | --- | --- | --- | --- | --- | --- | --- |
| **Percent undersampled^a^** | **Macro-F1** | **No SDoH** | **Employment** | **Housing** | **Parent** | **Relationship** | **Social Support** | **Transportation** |
| 10% |  |  |  |  |  |  |  |  |
| Gold data only | 0.586 | 0.996 | 0.745 | 0.333 | 0.000 | 0.947 | 0.484 | 0.600 |
| Gold + synthetic data | 0.654 | 0.996 | 0.809 | 0.600 | 0.452 | 0.919 | 0.566 | 0.235 |
| 25% |  |  |  |  |  |  |  |  |
| Gold data only | 0.638 | 0.997 | 0.750 | 0.571 | 0.160 | 0.969 | 0.516 | 0.500 |
| Gold + synthetic data | **0.659** | 0.996 | 0.822 | 0.545 | 0.452 | 0.938 | 0.571 | 0.286 |
| 40% |  |  |  |  |  |  |  |  |
| Gold data only | 0.534 | 0.995 | 0.719 | 0.000 | 0.087 | 0.932 | 0.604 | 0.400 |
| Gold + synthetic data | 0.638 | 0.996 | 0.810 | 0.667 | 0.400 | 0.956 | 0.455 | 0.182 |
| 50% |  |  |  |  |  |  |  |  |
| Gold data only | 0.500 | 0.995 | 0.683 | 0.000 | 0.083 | 0.957 | 0.559 | 0.222 |
| Gold + synthetic data | 0.686 | 0.996 | 0.824 | 0.750 | 0.465 | 0.935 | 0.629 | 0.200 |
| 70% |  |  |  |  |  |  |  |  |
| Gold data only | 0.449 | 0.995 | 0.657 | 0.000 | 0.167 | 0.900 | 0.425 | 0.000 |
| Gold + synthetic data | 0.607 | 0.994 | 0.720 | 0.545 | 0.400 | 0.825 | 0.429 | 0.333 |
| 75% |  |  |  |  |  |  |  |  |
| Gold data only | 0.493 | 0.996 | 0.746 | 0.000 | 0.240 | 0.933 | 0.533 | 0.000 |
| Gold + synthetic data | 0.596 | 0.995 | 0.782 | 0.500 | 0.074 | 0.886 | 0.537 | 0.400 |
| 90% |  |  |  |  |  |  |  |  |
| Gold data only | 0.183 | 0.988 | 0.000 | 0.000 | 0.000 | 0.259 | 0.031 | 0.000 |
| Gold + synthetic data | 0.505 | 0.994 | 0.733 | 0.400 | 0.143 | 0.849 | 0.108 | 0.308 |
| 100% |  |  |  |  |  |  |  |  |
| Gold data only | 0.00 | 0.00 | 0.00 | 0.00 | 0.00 | 0.00 | 0.00 | 0.00 |
| Gold + synthetic data | 0.063 | 0.000 | 0.047 | 0.017 | 0.228 | 0.036 | 0.095 | 0.015 |
| **Adverse SDoH** | | | | | | | | |
| **Percent undersampled^a^** | **Macro-F1** | **No SDoH** | **Employment** | **Housing** | **Parent** | **Relationship** | **Social Support** | **Transportation** |
| 10% |  |  |  |  |  |  |  |  |
| Gold data only | 0.535 | 0.997 | 0.746 | 0.400 | 0.083 | 0.933 | 0.143 | 0.444 |
| Gold + synthetic data | **0.671** | 0.997 | 0.719 | 0.750 | 0.474 | 0.921 | 0.375 | 0.462 |
| 25% |  |  |  |  |  |  |  |  |
| Gold data only | 0.548 | 0.997 | 0.643 | 0.571 | 0.000 | 0.881 | 0.143 | 0.600 |
| Gold + synthetic data | 0.646 | 0.997 | 0.716 | 0.667 | 0.378 | 0.879 | 0.353 | 0.533 |
| 40% |  |  |  |  |  |  |  |  |
| Gold data only | 0.565 | 0.998 | 0.730 | 0.000 | 0.410 | 0.933 | 0.286 | 0.600 |
| Gold + synthetic data | 0.578 | 0.997 | 0.635 | 0.545 | 0.400 | 0.903 | 0.300 | 0.267 |
| 50% |  |  |  |  |  |  |  |  |
| Gold data only | 0.471 | 0.997 | 0.688 | 0.571 | 0.000 | 0.755 | 0.000 | 0.286 |
| Gold + synthetic data | 0.641 | 0.997 | 0.690 | 0.667 | 0.313 | 0.921 | 0.400 | 0.500 |
| 70% |  |  |  |  |  |  |  |  |
| Gold data only | 0.408 | 0.997 | 0.500 | 0.000 | 0.160 | 0.808 | 0.143 | 0.250 |
| Gold + synthetic data | 0.566 | 0.997 | 0.646 | 0.600 | 0.389 | 0.929 | 0.154 | 0.250 |
| 75% |  |  |  |  |  |  |  |  |
| Gold data only | 0.309 | 0.996 | 0.256 | 0.000 | 0.000 | 0.410 | 0.000 | 0.500 |
| Gold + synthetic data | 0.534 | 0.996 | 0.480 | 0.545 | 0.294 | 0.866 | 0.273 | 0.286 |
| 90% |  |  |  |  |  |  |  |  |
| Gold data only | 0.142 | 0.995 | 0.000 | 0.000 | 0.000 | 0.000 | 0.000 | 0.000 |
| Gold + synthetic data | 0.426 | 0.996 | 0.311 | 0.462 | 0.160 | 0.747 | 0.143 | 0.167 |
| 100% |  |  |  |  |  |  |  |  |
| Gold data only | 0.00 | 0.00 | 0.00 | 0.00 | 0.00 | 0.00 | 0.00 | 0.00 |
| Gold + synthetic data | 0.040 | 0.000 | 0.009 | 0.007 | 0.227 | 0.011 | 0.015 | 0.011 |

Settings: FlanXL, guideline synthetic data OR no synthetic data.

^a^% undersampled means percent taken away (e.g., 25% undersampled = 25% of positive gold-labeled instances and 25% of negative gold-labeled instances removed).

SDoH = social determinants of health.

**Supplementary Table 2.** Most common discrepancies between ground-truth and best-performing model prediction for each task

| **Task** | **Ground Truth** | **Model Prediction** | **Count** |
| --- | --- | --- | --- |
| **Any SDoH Mention** | No SDoH | Support | 24 |
|  | No SDoH | Employment | 16 |
|  | Support | No SDoH | 10 |
| **Adverse SDoH Mention** | No SDoH | Employment | 12 |
|  | Parent | No SDoH | 10 |
|  | Employment | No SDoH | 6 |

SDoH = social determinants of health.

**Supplementary Table 3.** Confusion matrix for any SDoH mention gold label versus best-performing model prediction

|  |  | **Gold Label** | |
| --- | --- | --- | --- |
|  |  | Positive | Negative |
| **Any SDoH Model Prediction** | Positive | 89 | 3 |
|  | Negative | 4 | 58 |

SDoH = social determinants of health.

**Supplementary Table 4.** Confusion matrix for adverse SDoH mention gold label versus best-performing model prediction

|  |  | **Gold Label** | |
| --- | --- | --- | --- |
|  |  | Positive | Negative |
| **Adverse SDoH Model Prediction** | Positive | 45 | 13 |
|  | Negative | 3 | 93 |

SDoH = social determinants of health.

**Supplementary Table 5.** Confusion matrix for adverse SDoH gold label versus mapped Z-codes

|  |  | **Gold Label** | |
| --- | --- | --- | --- |
|  |  | Positive | Negative |
| **Mapped Z-codes** | Positive | 1 | 5 |
|  | Negative | 47 | 101 |

SDoH = social determinants of health.

**Supplementary Table 6.** Inter-annotator agreement for granular SDoH levels

| **Granular SDoH Label** | **Class-wise Krippendorff's (α)** |
| --- | --- |
| TRANSPORTATION_distance | 0.00 |
| TRANSPORTATION_resource | 0.67 |
| TRANSPORTATION_other | 0.00 |
| HOUSING_undomiciled | 0.00 |
| HOUSING_poor | 0.29 |
| HOUSING_other | 0.36 |
| RELATIONSHIP_married | 0.95 |
| RELATIONSHIP_partnered | 0.93 |
| RELATIONSHIP_divorced | 0.86 |
| RELATIONSHIP_widowed | 1.00 |
| RELATIONSHIP_single | 0.84 |
| PARENT | 0.81 |
| EMPLOYMENT_employed | 0.80 |
| EMPLOYMENT_underemployed | 0.40 |
| EMPLOYMENT_unemployed | 0.74 |
| EMPLOYMENT_disability | 0.90 |
| EMPLOYMENT_retired | 0.90 |
| EMPLOYMENT_student | 1.00 |
| SUPPORT_plus | 0.78 |
| SUPPORT_minus | 0.74 |

SDoH = social determinant of health.

**Supplementary Table 7.** Inter-annotator agreement for higher-level SDoH mention labels

| **Any SDoH Mention Label** | **Class-wise Krippendorff's (α)** |
| --- | --- |
| SUPPORT | 0.77 |
| EMPLOYMENT | 0.89 |
| HOUSING | 0.71 |
| TRANSPORTATION | 0.64 |
| PARENT | 0.81 |
| RELATIONSHIP | 0.95 |

SDoH = social determinant of health.

**Supplementary Table 8.** Prompts used to generate synthetic SDoH sentences using GPT3.5

| **Output Sentence Label^a^** | **Prompt** |
| --- | --- |
| Housing-Adverse | {"role": "system", "content": "You are a physician."},  {"role": "user", "content": "Examples of housing issues for patients: 1. Pt came from Assisted Living Corp. and complained about rent increase.\n2. “Pt came from Assisted Living Corp. and complained about rent increase.\n3. He says he is worried about making his mortgage payments.\n4. Pt is staying with a friend and does not have a mailing address.\n5. Pt currently staying at Barbara McInnis shelter.\n5. Pt is staying at the Motel for the time being, while on the waitlist for the Hope Lodge."},  {"role": "assistant", "content": "Ok I will remember that."},  {"role": "user", "content": "Imagine you are a physician. Please give me 100 sentences from your clinic notes about various patient's housing issues similar to the examples."} |
| Transportation-Adverse | {"role": "system", "content": "You are a physician."},  {"role": "user", "content": "Examples of transportation issues for patients: 1. Pt lives 30mi away from hospital and complains about needing to transfer three times each way.\n2. Pt missed appointment because her sister couldn't drive her today.\n3. Pt is worried about making appointments because the metro is under construction this month.\n4. Pt is worried about the two hour drive.\n5. She is having trouble lying flat for treatment, she thinks it is because her back hurts after the two hour car ride into clinic.\n6. Pt felt that coming to Los Angeles was hard for them and asked to be referred to Santa Cruz.\n7. He is having trouble getting to and from the hospital."},  {"role": "assistant", "content": "Ok I will remember that."},  {"role": "user", "content": "Imagine you are a physician. Please give me 100 sentences from your clinic notes about various patient's transportation issues similar to the examples."} |
| Relationship-Adverse | {"role": "system", "content": "You are a physician."},  {"role": "user", "content": "Examples of divorced, widowed, single, separated issues for patients: 1. Pt is meeting ex-wife at appointment.\n2. Pt is married but separated.\n3. Pt spouse passed away in October of last year.\n4. Pt is single.\n5. Pt arrived with his girlfriend, and his ex-wife will attend with him at next week’s session.\n6. Pt has 3 kids from former marriage"},  {"role": "assistant", "content": "Ok I will remember that."},  {"role": "user", "content": "Imagine you are a physician. Please give me 100 sentences from your clinic notes about various patients being divorced, widowed, single, or separated issues similar to the examples."} |
| Relationship-Not adverse | {"role": "system", "content": "You are a physician."},  {"role": "user", "content": "Examples of married/partnered sentences for patients: 1. Pt and her husband came into my office today.\n2. Pt and her fiancée came into my office today.\n3. He is here with his boyfriend.\n4. He is married to Sheila."},  {"role": "assistant", "content": "Ok I will remember that."},  {"role": "user", "content": "Imagine you are a physician. Please give me 100 sentences from your clinic notes about various patients being married / partnered similar to the examples."} |
| Parent-Adverse | {"role": "system", "content": "You are a physician."},  {"role": "user", "content": "Examples of parental status for patients: 1. Pt has 2 children ages 9 and 13.\n 2. Pt has 2 teenage children.\n3. Pt was seen today with his daughter Angela, 3 y/o for a routine checkup."},  {"role": "assistant", "content": "Ok I will remember that."},  {"role": "user", "content": "Imagine you are a physician. Please give me 100 sentences from your clinic notes about various patients being a parent to minors similar to the examples."} |
| Employment-Adverse | {"role": "system", "content": "You are a physician."},  {"role": "user", "content": "Examples of employment issues for patients: 1. Pt works part-time at Jim's Fish and is struggling to pay rent.\n2. Pt has been living off of unemployment for the past 2 months.\n3. Used to be a car mechanic, but he has been on disability for the past 2 years since his diagnosis.\n4. He is currently on disability and is also occasionally working as an Uber driver to help cover the bills."},  {"role": "assistant", "content": "Ok I will remember that."},  {"role": "user", "content": "Imagine you are a physician. Please give me 100 sentences from your clinic notes about various patient's employment issues similar to the examples."} |
| Employment-Not adverse | {"role": "system", "content": "You are a physician."},  {"role": "user", "content": "Examples of employment sentences for patients: 1. Pt works as an electrician in Rockland.\n2. Pt is a 75yr old retiree.\n3. Pt is attending Cool University full time.\n4. Pt is a semi-retired marketing consultant."},  {"role": "assistant", "content": "Ok I will remember that."},  {"role": "user", "content": "Imagine you are a physician. Please give me 100 sentences from your clinic notes about various patient's employment similar to the examples."} |
| Social support-Adverse | {"role": "system", "content": "You are a physician."},  {"role": "user", "content": "Examples of social support issues for patients: 1. Pt lives alone.\n2. Pt is struggling to find someone to watch his cat on the days he has to come for treatment."},  {"role": "assistant", "content": "Ok I will remember that."},  {"role": "user", "content": "Imagine you are a physician. Please give me 100 sentences from your clinic notes about various patient's lack of social support similar to the examples."} |
| Social support-Not adverse | {"role": "system", "content": "You are a physician."},  {"role": "user", "content": "Examples of social support sentences for patients: 1. Here today is Pt, her daughter, and supportive wife.\n2. Pt is living with his parents during treatment, while his neighbors watch his cat.\n3. Pt had to borrow money from her friend to catch the bus today.\n4. Pt is currently living with nephew while receiving treatment."},  {"role": "assistant", "content": "Ok I will remember that."},  {"role": "user", "content": "Imagine you are a physician. Please give me 100 sentences from your clinic notes about various patient's social support similar to the examples."} |

^a^Output sentence label is the label assigned to all synthetic sentences generated from the prompt.

**Supplementary Table 9.** Z-Code to SDoH label mappings

| **Z-Code** | **SDoH label mapping** |
| --- | --- |
| Z55: Education and literacy | EDUCATION_none |
| Z56: Employment and unemployment | EMPLOYMENT_unemployed,  EMPLOYMENT_underemployed, EMPLOYMENT_employed (with adverse work environment) |
| Z59: Housing and economic circumstances | HOUSING, SUPPORT_minus, EMPLOYMENT_un(der)employed |
| Z60: Social environment | SUPPORT_minus |
| Z62: Upbringing | PARENT, SUPPORT_minus |
| Z63: Other problems related to primary support group, including family circumstances | SUPPORT_minus |
| Z75: Problems related to medical facilities and other health care | HOUSING, TRANSPORTATION |

SDoH = social determinant of health.

**Supplementary Figures**


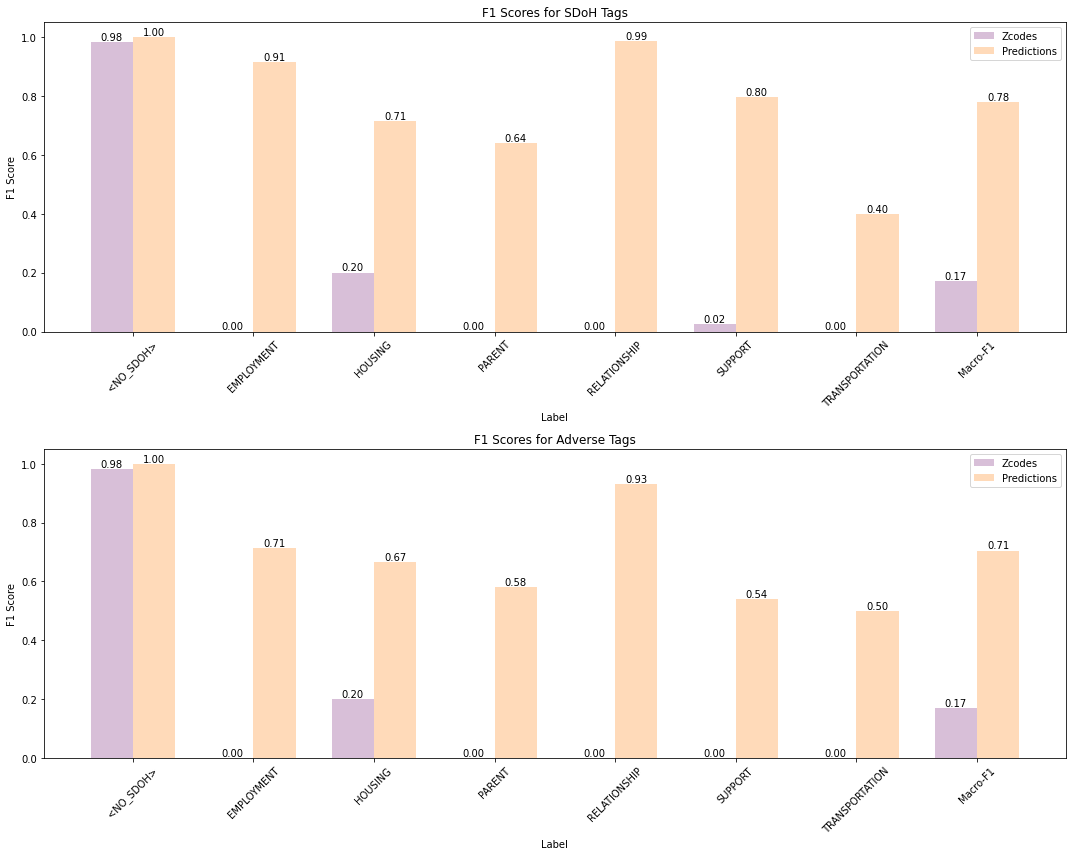


**Supplementary Figure 1.** Class-wise and Macro-F1 scores of our best-performing model against mapped Z-Codes at the patient level (on test set and dev set).


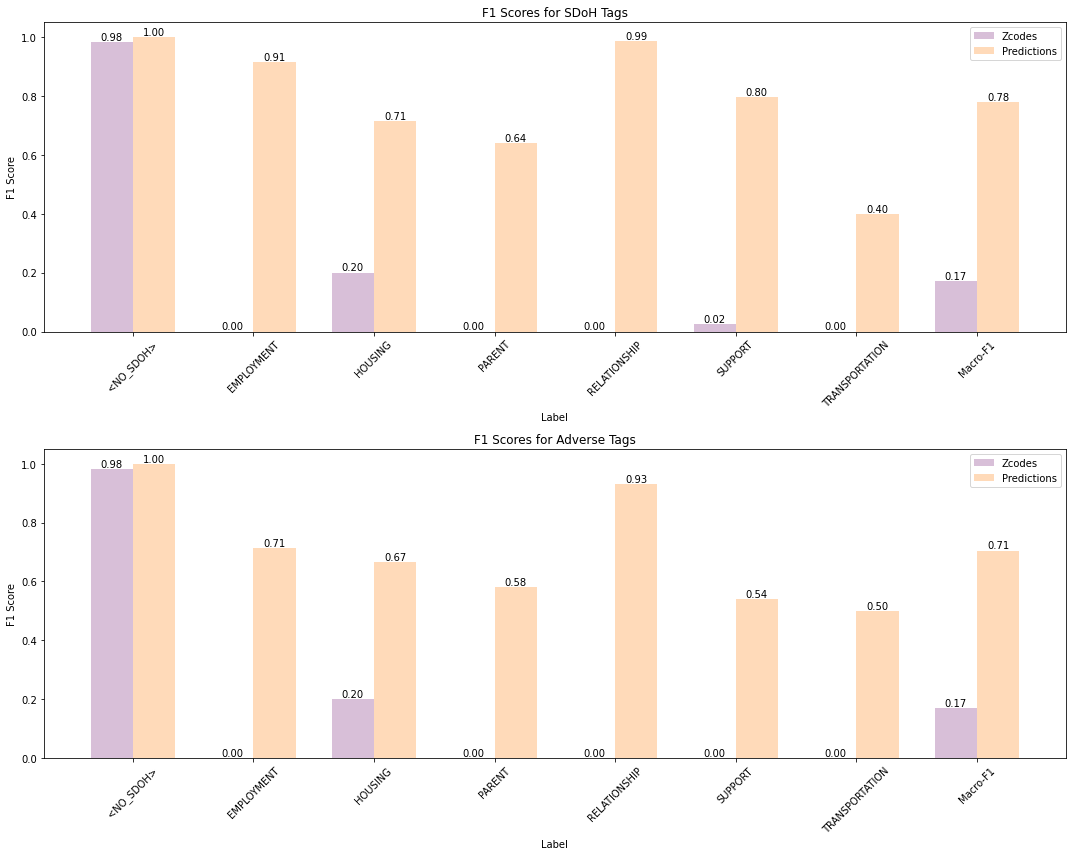
 **Supplementary Figure 2.** Class-wise and Macro-F1 scores of our best-performing model against mapped Z-Codes at the patient level (on test set only).
